# Combined central retinal artery and vein occlusion following trabeculectomy

**DOI:** 10.3205/oc000205

**Published:** 2022-06-28

**Authors:** Albert John Bromeo, Sweet Jorlene Lerit, Patricia Grulla-Quilendrino, George Michael Sosuan, Edgar Leuenberger

**Affiliations:** 1 Asian Eye Institute, Makati, Philippines

**Keywords:** retinal artery occlusion, retinal vein occlusion, glaucoma, trabeculectomy

## Abstract

Retinal vascular events may occur as rare complications of glaucoma procedures due to various factors, including exacerbation of ischemia in patients with pre-existing vascular comorbidities, toxic effect of mitomycin-C, and decompression retinopathy. We present the case of a 47-year-old hypertensive male who underwent trabeculectomy for advanced glaucoma in his right eye. At 3 weeks postoperatively, he presented with a drop in visual acuity to light perception with a spike in intraocular pressure. On examination, there was increased bleb vascularity as well as rubeosis. Fundoscopy revealed findings consistent with both central retinal artery occlusion and central retinal vein occlusion. Combined central retinal artery and vein occlusion is a rare retinal vascular condition. Neovascular glaucoma can occur as a sequelae of the ischemic process in the retina. Despite treatment, there is a poor visual prognosis, with the affected eye usually becoming blind from optic atrophy and neovascularization.

## Introduction

Retinal vascular occlusions after intraocular procedures are rare. Various forms of retinal artery and vein occlusions have been reported following trabeculectomy. The exact mechanism behind their occurrence is unknown, but it is theorized that intraoperative pressure changes occurring intraoperatively and postoperatively act as a trigger for vascular events, particularly in patients with predisposing cardiovascular conditions [1].

Combined central artery and vein occlusion (CCRAVO) is a particularly rare retinal vascular condition combining the clinical features of central retinal artery occlusion (CRAO) and central retinal vein occlusion (CRVO). Only a small number of cases have been reported, thus the clinical course and management have not been well established. CCRAVO has been reported in association with many conditions, including systemic lupus erythematosus, orbital inflammatory disease, Behcet’s syndrome, posterior scleritis, optic neuritis, leukemia, lymphoma, homocysteinemia, retrobulbar injection, and trauma. The condition is usually associated with a poor visual prognosis [2].

To the best of our knowledge, this is the first reported case of CCRAVO occurring as a complication of glaucoma filtering surgery, highlighting the diagnosis and management of this rare complication. 

## Case description

**Figure 1:**
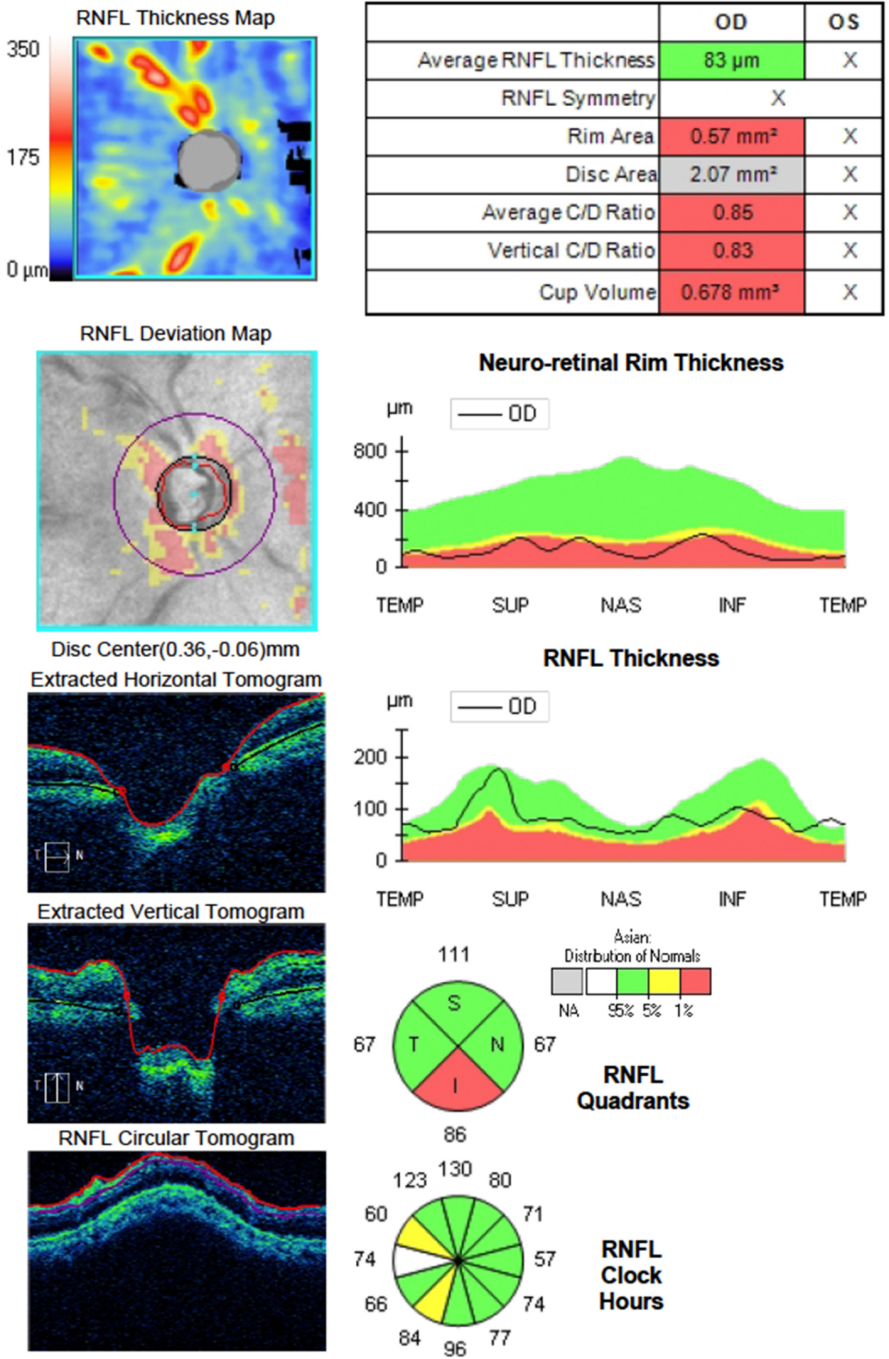
Preoperative optical coherence tomography of the optic nerve head showing cupping and thinning of the neuroretinal rim at its inferior aspect

A 47-year-old male with a prior history of hypertension was being managed as a known case of juvenile open angle glaucoma on both eyes. The best corrected visual acuity (BCVA) on the right eye was hand motions, with an intraocular pressure (IOP) of 26 mmHg and optic disc examination and perimetry results consistent with advanced glaucoma. The left eye had a vision of no light perception from absolute glaucoma. Preoperative eye examination was significant for glaucomatous optic neuropathy with advanced neuroretinal rim thinning and disc pallor, as demonstrated by optical coherence tomography (OCT) of the optic nerve head (Figure 1 (Fig. 1)). The anterior segment and the rest of the retina was essentially normal. The low vision did not permit visual field testing. The patient underwent trabeculectomy with mitomycin-C in the right eye. The surgery and the initial postoperative course was unremarkable. The IOP in the initial postoperative period was at target and maintained within the low teens.

**Figure 2:**
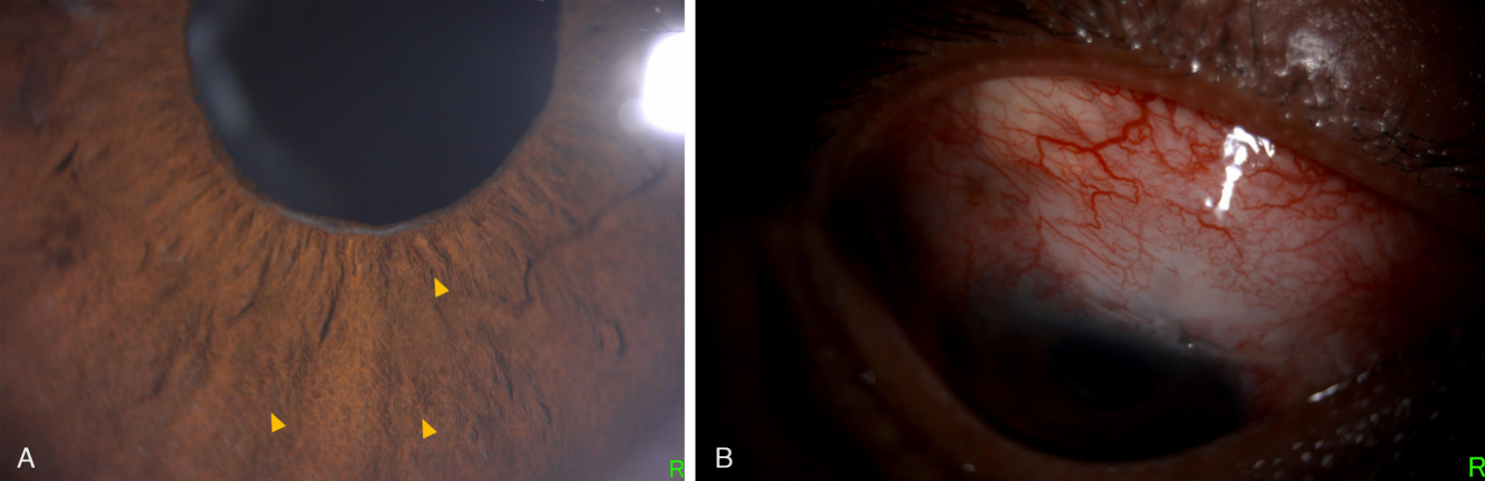
Slit lamp photographs showing (A) fine rubeosis affecting the pupil edge and iris (yellow arrowheads) and (B) low rise and increased vascularity on the filtering bleb

**Figure 3:**
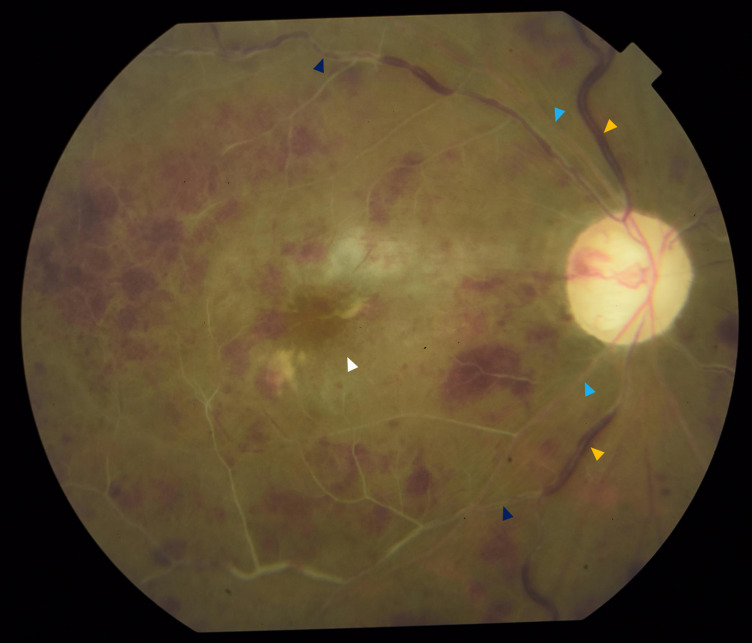
Fundus photograph showing features consistent with both CRAO and CRVO, including dilated and tortuous veins (yellow arrowhead), ischemic retinal whitening with a cherry red spot (white arrowhead), and sclerosed arteries (light blue arrowhead), as well as sclerosed veins with boxcarring of red blood cells (dark blue arrowhead)

At 3 weeks postoperatively, the patient presented with decreased vision in the right eye. The BCVA dropped to light perception. On biomicroscopy, fine rubeosis on the pupil edge, iris surface, and angles were identified (Figure 2A (Fig. 2)). There was a low bleb with increased vascularity (Figure 2B (Fig. 2)). The IOP spiked to 33 mmHg. Fundoscopy revealed dilated and tortuous retinal veins, sclerosed retinal arteries and veins with boxcarring of red blood cells, numerous intraretinal hemorrhages, and retinal ischemic whitening with a cherry red spot – findings consistent with both CRAO and CRVO (Figure 3 (Fig. 3)).

**Figure 4:**
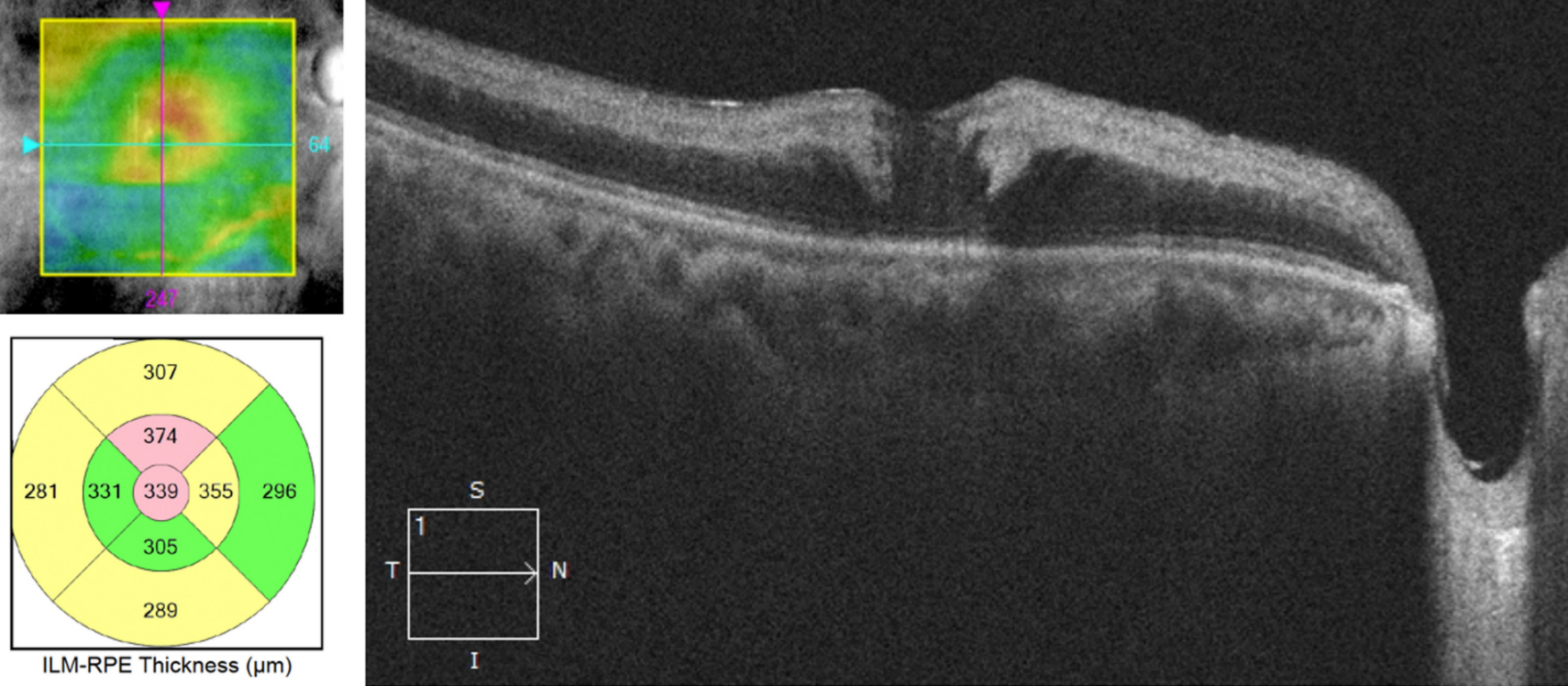
Optical coherence tomography scan showing hyperreflective and thickened inner retinal layers that correspond to retinal edema as well as mild cystoid changes in the fovea

Optical coherence tomography (OCT) showed increased reflectivity and thickness of the inner retinal layers with posterior shadowing on the outer retinal layers. There was also minimal cystoid macular edema at the fovea (Figure 4 (Fig. 4)).

**Figure 5:**
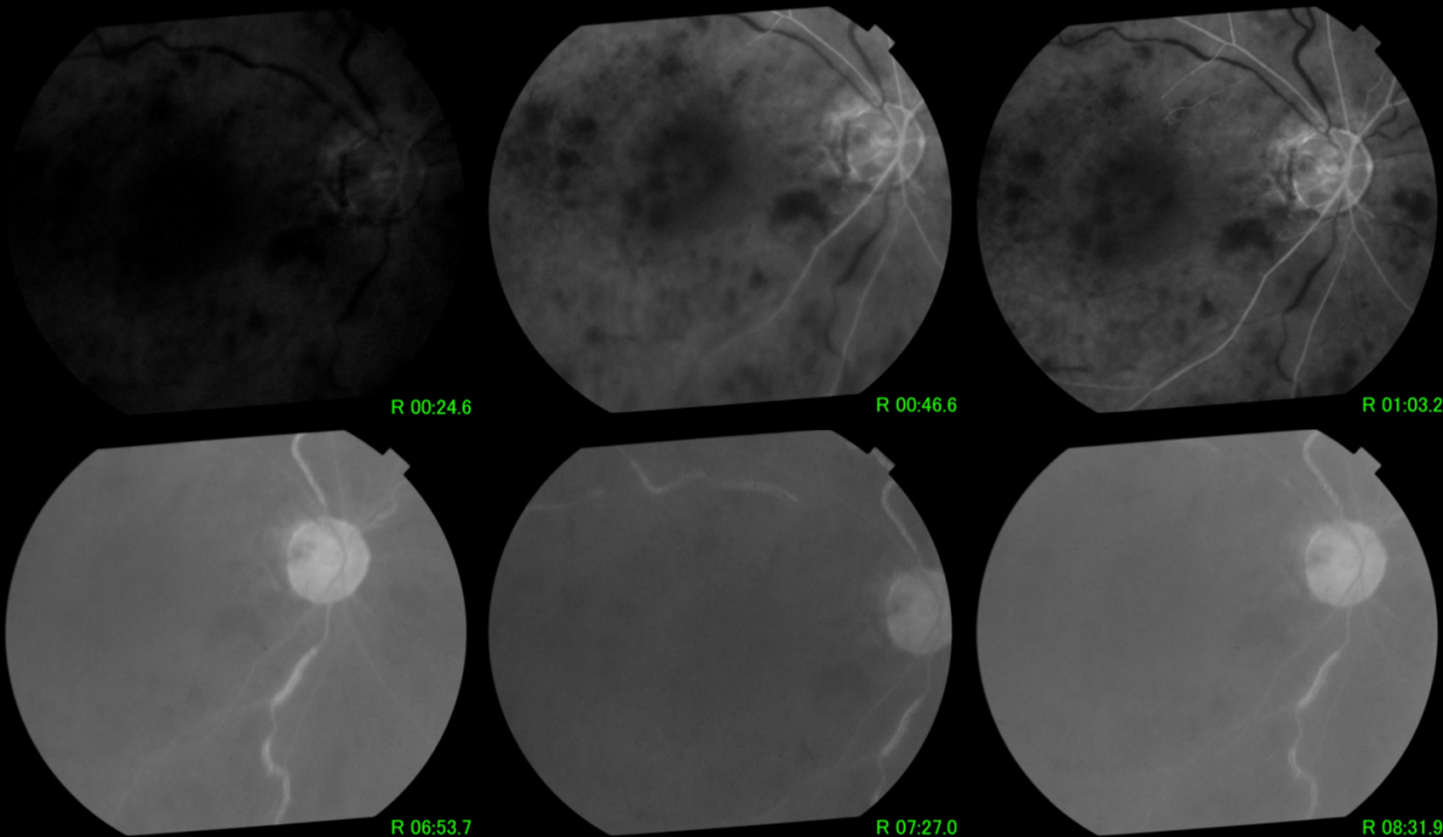
Fluorescein angiogram showing delayed filling of arteries, increased arteriovenous transit time with subsequent delay in venous filling, widespread capillary nonperfusion, and minimal macular edema

Fundus fluorescein angiography (FFA) done at presentation (Figure 5 (Fig. 5)) showed delayed filling of the retinal arteries with an arm-to-retina time of 46.6 seconds. There was a corollary increase in arteriovenous transit time with delayed filling of the retinal veins, with the first fill noted at 6 minutes and 53 seconds. There was widespread capillary nonperfusion and numerous areas of blocked hypofluorescence from the intraretinal hemorrhages. There was minimal leakage on the macula on FFA.

The clinical features as well as results of the FFA and OCT confirmed the diagnosis of CCRAVO with secondary neovascular glaucoma.

**Figure 6:**
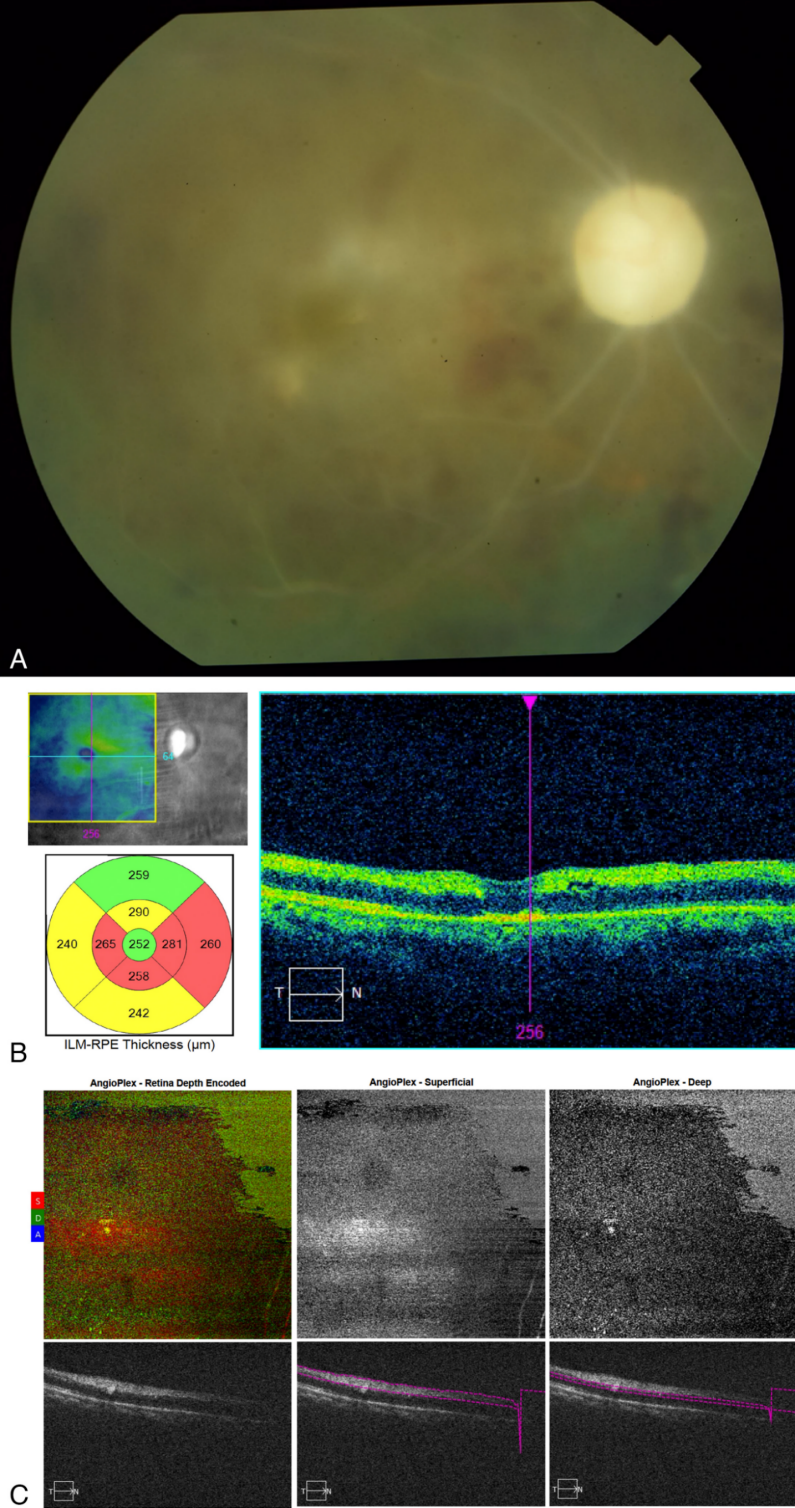
Multimodal imaging after 3 months of treatment; (A) fundus photograph showing optic atrophy and sclerosis of all retinal vessels with residual intraretinal hemorrhages; (B) optical coherence tomography scan showing generalized retinal atrophy more prominent in the inner retinal layers; (C) optical coherence tomography angiography showing absence of blood flow within the retinal vessels in both the superficial and deep capillary plexuses

Panretinal photocoagulation (PRP) as well as intravitreal injection of anti-VEGF (bevacizumab) was promptly done. The patient followed up after 1 week with resolution of rubeosis and lowering of IOP back to target levels. After 1 month, repeat ophthalmologic evaluation showed persistence of minimal macular edema, and the patient was given another intravitreal injection of bevacizumab. Subsequent follow-up examinations showed stable eye findings. At the time of the last visit (3 months following treatment), BCVA stabilized at hand motions, anterior segment findings were unremarkable, and IOP maintained at the low teens. Fundoscopy showed widespread retinal atrophic changes with sclerosis of all retinal vessels, as well as optic atrophy (Figure 6 (Fig. 6)). There were no signs of iris, disc, or retinal neovascularization.

The patient was referred back to his cardiologist for cardiovascular risk assessment, particularly carotid evaluation. Carotid ultrasound with Doppler scan was unremarkable. The patient was advised to continue oral medications for hypertension.

## Discussion

Retinal vascular occlusions can occur as a complication of trabeculectomy, particularly in patients with coexisting cardiovascular diseases [1]. It is theorized that there is a shift in the lamina cribrosa from perioperative IOP changes which act as a triggering mechanism that contributes to occlusion of the retinal vasculature [3]. Several other etiologies have been postulated, including exacerbation of ischemia from pre-existing vascular comorbidities, diffusion of mitomycin-C into the vitreous and development of retinal toxicity, and decompression retinopathy [1], [4].

CCRAVO is a rare vascular event. The diagnosis is based on ophthalmoscopic fundus findings which combines the features of both CRAO and CRVO, including tortuosity and dilation of the retinal veins, swelling and hyperemia of the optic disc, intraretinal hemorrhages, superficial ischemic retinal whitening with a cherry-red spot, cotton wool spots, or Roth spots [2]. The exact mechanism of CCRAVO has not been established, although it is theorized that CRVO plays a more pivotal role in the pathophysiology than CRAO. It is postulated that the CRAO seen in CCRAVO may not be a true CRAO but occurs as a consequence of the occlusion of the central retinal vein at the level of the lamina cribrosa. The occlusion of the retinal venous drainage increases the intraluminal pressure in the retinal veins which is then transmitted into the retinal arteries, compromising entry of blood into the eye, resulting in a CRAO-like condition. Previous reports also show that CRVO persists much longer than CRAO in affected eyes [2], [5].

Rubeosis iridis develops in 81% of eyes, leading to neovascular glaucoma. This can be seen as early as 1–2 weeks, but on average at about 6 weeks [5]. Previous reports show that the visual damage is much worse in those that develop neovascularization in the anterior segment, suggesting more severe ischemic damage in these eyes. The mechanism of neovascularization in CCRAVO is theorized to be similar to that of CRAO, which is associated with prolonged retinal vessel filling and failure of return of reperfusion. Due to the limited number of reported cases of CCRAVO, data on the incidence of neovascular glaucoma following CCRAVO is limited [2].

The management of CCRAVO is likewise not well established. In eyes with neovascularization, aggressive treatment with PRP is recommended [2]. However, the visual prognosis in these eyes is generally dismal despite adequate treatment. There is usually failure to gain retinal reperfusion in these cases.

In the case presented, CCRAVO occurred in an eye that already had a poor visual prognosis due to advanced glaucoma. Prompt treatment with PRP and intravitreal anti-VEGF managed to control the neovascularization and lower IOP, with return to baseline BCVA. However, continuous follow-up and monitoring are still needed in these cases. CCRAVO is usually associated with sequelae such as optic atrophy, neovascularization, and vitreous hemorrhage [6].

This case presented the diagnosis and management of CCRAVO occurring as a complication of glaucoma filtering surgery. While there is literature discussing the mechanisms behind retinal vascular events occurring after glaucoma filtering surgery, it should be noted that the relationship between CCRAVO and trabeculectomy in this case may not be causal and is a limitation of this case report.

## Conclusion

Retinal vascular occlusions can occur as an uncommon complication of glaucoma filtering surgery, particularly in patients with pre-existing cardiovascular conditions. Combined central retinal artery and vein occlusion (CCRAVO) is a rare retinal vascular condition which combines the features of both central retinal vein occlusion (CRVO) and central retinal artery occlusion (CRAO). CCRAVO can lead to rubeosis iridis and eventually secondary neovascular glaucoma. Prompt panretinal photocoagulation is recommended. Despite aggressive treatment, the visual prognosis in eyes with CCRAVO is usually poor.

## Notes

### Competing interests

The authors declare that they have no competing interests.
